# Usefulness and limitations of the acute respiratory distress syndrome definitions in non-intubated patients. A narrative review

**DOI:** 10.3389/fmed.2023.1088709

**Published:** 2023-02-23

**Authors:** Martin Zbiral, Maximilian Weber, Sebastian König, Felix Kraft, Roman Ullrich, Katharina Krenn

**Affiliations:** ^1^Department of Anesthesia, General Intensive Care and Pain Medicine, Medical University of Vienna, Vienna, Austria; ^2^Department of Anesthesiology and Intensive Care Medicine, AUVA Trauma Center Vienna, Vienna, Austria

**Keywords:** hypoxia, non-invasive ventilation, high-flow nasal cannula, high-flow nasal oxygen, acute respiratory failure, Berlin definition, acute respiratory distress syndrome

## Abstract

According to the Berlin Definition of acute respiratory distress syndrome (ARDS), a positive end-expiratory pressure (PEEP) of at least 5 cmH_2_O is required to diagnose and grade ARDS. While the Berlin consensus statement specifically acknowledges the role of non-invasive ventilation (NIV) in mild ARDS, this stratification has traditionally presumed a mechanically ventilated patient in the context of moderate to severe ARDS. This may not accurately reflect today’s reality of clinical respiratory care. NIV and high-flow nasal cannula oxygen therapy (HFNO) have been used for managing of severe forms of acute hypoxemic respiratory failure with growing frequency, including in patients showing pathophysiological signs of ARDS. This became especially relevant during the COVID-19 pandemic. The levels of PEEP achieved with HFNO have been particularly controversial, and the exact FiO_2_ it achieves is subject to variability. Pinpointing the presence of ARDS in patients receiving HNFO and the severity in those receiving NIV therefore remains methodically problematic. This narrative review highlights the evolution of the ARDS definition in the context of non-invasive ventilatory support and provides an overview of the parallel development of definitions and ventilatory management of ARDS. It summarizes the methodology applied in clinical trials to classify ARDS in non-intubated patients and the respective consequences on treatment. As ARDS severity has significant therapeutic and prognostic consequences, and earlier treatment in non-intubated patients may be beneficial, closing this knowledge gap may ultimately be a relevant step to improve comparability in clinical trial design and outcomes.

## 1. Introduction

In pathophysiological terms, acute respiratory distress syndrome (ARDS) is an inflammatory reaction of the lung to certain triggers (1). It results in loss of integrity of the alveolo-capillary barrier with consecutive protein-rich exudates causing non-hydrostatic pulmonary edema and inactivation of surfactant. The inflammation is sustained and propagated by a complex immunologic interplay and eventually progresses to either recovery, various degrees of chronic fibrotic lung damage, or even death.

The clinical features of ARDS are those of type 1 respiratory failure, namely profound, refractory hypoxia paired with bilateral opacities in chest x-ray (CXR) or computed tomography (CT). Of note, even in the original description by David Ashbaugh and colleagues, this acute hypoxemic respiratory failure (AHRF) is neither in proportion to nor a consequence of coexisting prior lung disease, congestive heart failure, or fluid overload (2). In fact, it may occur in the absence of any of the mentioned factors. Since Ashbaugh’s report, several definitions have been proposed to characterize the phenotype of ARDS and standardize its diagnosis, treatment, and severity stratification for research (Table 1). The currently used Berlin Definition of ARDS was published in 2012 and has replaced the American-European Consensus Conference (AECC) definition (3–5).

**Table 1:** Overview of the definitions for acute respiratory distress syndrome (ARDS).

ARDS-definition	Author	Year	Timing	Chest imaging for detection of alveolar infiltrates	Oxygenation	Categories/Subgroups	Other criteria
First description of ARDS	Ashbaugh et al. (2)	1967		Chest radiograph	Arterial hypoxemia refractory to oxygen or ventilation therapy		Decreased respiratory system compliance
Lung injury score	Murray et al. (3)	1988	Sudden onset or rapid progression	Chest radiograph scored for number of quadrants with infiltrates (1–4)	PaO_2_/FiO_2_ < 300	Differentiates between mild to-moderate and severe lung injury (ARDS) depending on score value	PEEP >5 cmH_2_O, if applied; respiratory system compliance <80 ml/cmH_2_O, when available
AECC definition	Bernard et al. (5)	1994	Acute onset	Frontal chest radiograph, infiltrates have to be bilateral	PaO_2_/FiO_2_ < 300 mmHg (regardless of PEEP)	Differentiates between acute lung injury and ARDS depending on PaO_2_/FiO_2_-ratio	No increased PAWP or no clinical evidence of left ventricular failure (exclusion of cardiogenic pulmonary edema)
Berlin definition	The ARDS definition task force, Ranieri et al. (4)	2012	Within 1 week of a known trigger or onset or deterioration of respiratory symptoms	CT scan or chest radiograph, infiltrates have to be bilateral and radiological picture must not be fully explained by other thoracic pathologies	PaO_2_/FiO_2_ ≤ 300 mmHg (with PEEP or in mild ARDS CPAP ≥5 cmH_2_O)	Differentiates between mild, moderate, and severe ARDS depending on PaO_2_/FiO_2_-ratio	Symptoms “not fully explained” by cardiogenic pulmonary edema or fluid retention

PaO_2_, arterial partial pressure of oxygen; FiO_2_, fraction of inspired oxygen; PEEP, positive end-expiratory pressure; AECC, American European Consensus Conference; PAWP, pulmonary arterial wedge pressure; CT, computer tomography; CPAP, continuous positive airway pressure.

## 2. Ventilatory support in acute hypoxemic respiratory failure

### 2.1. Non-invasive ventilatory support

In clinical practice, any type of respiratory failure is commonly managed with an escalating sequence of respiratory support methods, including conventional supplemental oxygen therapy, various types of non-invasive ventilatory support, and, if the aforementioned strategies and pharmaceutical treatments, such as antibiotics or bronchodilators, fail to improve the patient’s condition, endotracheal intubation and mechanical ventilation (6, 7). Non-invasive ventilation (NIV) has been proposed as an early intervention in mild ARDS to reduce the rate of intubation, ventilator associated pneumonia, and length of intensive care unit (ICU) stay (8). NIV can be administered *via* a tight-fitting facemask as well as a helmet, each entailing their own advantages and disadvantages (9). Helmet NIV may be associated with reduced mortality and a lower rate of intubation according to one meta-analysis, although the included trials analyzed were small in size (10).

Not all guidelines deem the current – in some respects equivocal – evidence sufficient to recommend NIV in new-onset AHRF (11). Just as patients may benefit from avoidance of intubation by successful non-invasive respiratory care, a delay in intubation by prolonged attempts of NIV as well as NIV in patients with a P_a_O_2_/F_i_O_2_ ratio of less than 150 may be harmful (12, 13). However, a recent meta-analysis has challenged this assumption in the context of COVID-19, as it found no difference in mortality between early and late intubation (14). AHRF with a P_a_O_2_/F_i_O_2_ ratio of 146 or less, a high expiratory minute volume as well as ARDS have been reported to be independent risk factors for NIV failure, defined as the need for endotracheal intubation, although the exact clinical triggers for this decision varied among protocols (15, 16). Interestingly, in one trial during the earlier phase of the pandemic, COVID-19 patients had an increased risk of NIV failure compared to matched non-COVID-19 ARDS patients (17). Strenuous respiratory efforts in patients receiving NIV for a prolonged time prior to intubation have also been linked to a phenomenon termed patient self-inflicted lung injury (P-SILI), which may further contribute to worse outcomes (18, 19).

In recent years, high flow nasal oxygen (HFNO) therapy has emerged as an effective and well-tolerated tool of post-extubation respiratory care (20, 21) and treatment of respiratory failure (22). Warmed, humidified oxygen is delivered to the patient at a flow rate that may match or considerably exceed peak inspiratory flow rates, reducing the work of breathing. In addition, HFNO leads to dead space washout, improving decarboxylation. It may create a small positive end-expiratory airway pressure (PEEP) in the order of 2–7 cmH_2_O, but the exact magnitude of this effect is still subject to debate and may depend on the gas flow as well as the caliber of cannulas, and may be significantly decreased if the subject opens their mouth (23–25).

High flow nasal oxygen has been suggested to reduce the rate of endotracheal intubation in both COVID-19-associated and non-COVID-19-associated AHRF in several studies and guidelines (26–29). One trial also suggested lower mortality rates with HFNO compared to conventional oxygen therapy and NIV, although this result was not assessed as the primary outcome (30), and was put into question by a recent Cochrane review (31). Nonetheless, current guidelines recommend the use of HFNO as a first-line therapy for acute type 1 respiratory failure, and guidelines of the European Respiratory Society have explicitly recommended HFNO over NIV and conventional oxygen therapy in these patients (11, 32, 33).

Immunocompromised patients represent a specific patient population, in whom treatment with NIV for AHRF plays an important role. In these patients, treatment with NIV is more frequently applied than in other patients with AHRF (34). This may partially be explained by evidence suggesting that immunocompromised patients requiring invasive mechanical ventilation (MV) have a higher mortality (35). In addition to recent evidence, findings suggest that especially the use of HFNO may significantly reduce the rate of intubation in immunocompromised patients with AHRF (36). However, there may be a downside to this trend, as delaying intubation in an immunocompromised patient who ultimately requires invasive MV is associated with a higher mortality (37). Therefore, correct assessment of disease severity seems to be of utmost importance in in this special patient population as to not delay necessary treatment decisions.

### 2.2. Invasive mechanical ventilation

Guidelines and strategies for invasive MV have evolved over the past decades (6). The current standard of care focuses on the avoidance of ventilator associated lung injury, using a bundle of care termed lung protective ventilation (38). This involves limiting tidal volumes to 6 ml/kg predicted body weight, avoiding plateau pressures (*P*_plat_) beyond 30 cmH_2_O, low driving pressure, i.e., the difference between PEEP and *P*_plat_ (39), and applying a higher level of PEEP in moderate to severe ARDS (40). In recent years, the COVID-19 pandemic and the associated paucity of resources has shifted clinical and academic interest to non-invasive ventilatory support even in more severe cases of AHRF.

## 3. Aspects of the Berlin definition

An early diagnosis of ARDS in patients with respiratory failure may lead to timely initiation of evidence-based measures to improve outcomes and has been recommended in clinical guidelines (6). This led us to review the respective aspects of the definition.

### 3.1. Timing

“Acute” is commonly defined as onset within 1 week after exposure to a clinical risk factor or “new or worsening respiratory symptoms” (4). The main differences to the predecessor definition of the AECC are the specification of a period and the requirement of a risk factor. In certain conditions, the onset of respiratory symptoms may precede ARDS far more than 1 week. Early variant COVID-19 infections have shown a tendency to delayed clinical deterioration, with a time to ICU admission and ARDS diagnosis exceeding 1 week of symptoms in a significant proportion of patients (41). In addition, patients with COVID-19 or other underlying diseases with a duration of ICU stay and mechanical ventilation for more than 1 week may experience protracted episodes of respiratory failure. Causes may be bacterial or fungal superinfections and ventilator-associated pneumonia that only eventually fulfill all ARDS criteria at the same time.

### 3.2. Chest imaging

The diagnosis of ARDS requires the presence of bilateral opacities on CXR or, in contrast to the AECC criteria, in a CT scan (4). However, patients presenting with bilateral opacities of only two quadrants and thereby qualifying for ARDS showed similar outcomes as AHRF patients with opacities of two unilateral quadrants in a large observational study (42). Although a requirement for more extensive opacities was discussed for severe ARDS, these considerations have been dismissed.

Lung ultrasound (LUS) has gained increased availability and frequency of routine use. A growing body of literature supports its application, a development already observed in the 2009 influenza pandemic (43) and further accelerated by the COVID-19 pandemic. LUS-based protocols have been proposed as an adjunct measure for the diagnosis and monitoring of ARDS in emergency departments (44) and ICUs (45). A frequently quoted systematic approach is the LUS score, ranging from 0 (normal lung) to 36 (severe lung injury) (46, 47). It is a semi-quantitative measure of severity of lung injury based on the presence of B-lines, pleural line abnormalities, and consolidations assessed in 12 defined anatomical regions. In a retrospective study in COVID-19 patients comparing a modified LUS protocol and chest CT, scores <13 and >23 were shown to have high sensitivity and specificity for mild and severe disease, respectively, whereas scores in the mid-range provided less diagnostic information (48). The latter observation as well as limitations inherent to the technique (i.e., diagnostic value may be influenced by operator experience, available time, etc.) may pose obstacles to the widespread implementation of LUS in incipient ARDS. The operator learning curve has been described variably. While some have suggested a steep learning curve, requiring a practitioner to perform as few as a dozen of examinations to gain sufficient skill, other experts are of the opinion that true competence may not be attained in a short time (49). However, the fact that LUS has been shown to predict mortality may still warrant its consideration in future ARDS definitions (50). Because of increased routine use of LUS, ICU patients may not undergo routine CXR as often (51), and LUS may be more readily available in low resource settings, where access to high end radiological devices may be limited (52).

### 3.3. Origin of edema

Prior to Ashbaugh’s landmark description of what is today known as ARDS, a condition termed “congestive atelectasis” with features including tachypnea, hypoxemia, a “stiff” lung and alveolar collapse was described in patients who had received large quantities of intravenous fluids and transfusions (53). Some authors consider this phenomenon a predecessor of ARDS. Indeed, cardiogenic pulmonary edema and fluid overload are frequent differential diagnoses of ARDS. In contrast to these early descriptions, both the AECC and the Berlin Definition require that the etiology of respiratory failure should not be “fully explained” by those conditions (4, 54). The current definition also no longer calls for measuring pulmonary artery wedge pressure. However, even in the absence of a clinical risk factor, it is necessary to objectively rule out hydrostatic edema. This process may be assisted by bedside echocardiography or different methods of cardiac output measurement, but has not yet been strictly defined. This may be due to concerns about a diagnostic delay caused by awaiting the availability of expert echocardiographic measurements or cardiac output measurement devices. In addition, patients with transient fluid overload and oxygenation perturbations persisting only for a few hours with subsequent rapid clinical improvement may not be the target population for therapeutic clinical trials in ARDS, so that certain clinical study protocols require persistence of impaired oxygenation for a specified period prior to randomization (e.g., NCT03567577: clinical stability for 8 h and NCT04417036: persistence of a P_a_O_2_/F_i_O_2_ ratio < 300 for a minimum of 4 h).

### 3.4. Oxygenation

The Berlin Definition uses the ratio of P_a_O_2_/F_i_O_2_ to stratify ARDS severity into mild, moderate and severe, with ratios of 300–201, 200–101, and 100 or less, respectively (4). Apart from the partially refined criteria, as compared to the AECC definition, and eliminating the term “acute lung injury (ALI)” for mild ARDS, a key requirement for diagnosis is a minimum PEEP or, in mild ARDS, continuous positive airway pressure (CPAP), of 5 cmH_2_O. The rationale was that the P_a_O_2_/F_i_O_2_ ratio may vary according to ventilator settings, particularly PEEP (54).

As discussed previously, respiratory failure is initially managed with non-invasive interventions in clinical practice. This is a challenge to the effort to validly diagnose, categorize, and study ARDS for several reasons. In non-intubated patients, there is no definitive consensus on the PEEP achieved with HFNO, which has been recommended for managing mild and moderate ARDS (55). The median end expiratory nasopharyngeal pressure achieved with HFNO in healthy volunteers with closed mouths using flow rates of 40 and 60 l/min has been reported as 3.6 and 6.8 cmH_2_O, respectively (24). Opening the mouth virtually nullified this effect. Of note, using CPAP set to 4 cmH_2_O achieved a median nasopharyngeal pressure of 3.1 cmH_2_O.

Furthermore, Riviello and colleagues highlighted diagnostic limitations in low-resource countries (52). The possible scarcity of available blood gas analyzers and mechanical ventilators may be overcome by use of their Kigali modification of the Berlin criteria, which omits the PEEP criterion and assesses oxygenation using an SpO_2_/FiO_2_ index. Several SpO_2_-based indices have been proposed to that end. Such indices may facilitate and expedite ARDS diagnosis, correlate to P_a_O_2_/F_i_O_2_ ratio, especially if PEEP is applied, and have even been suggested by some authors to be predictive of outcomes (56–58). Nevertheless, FiO_2_ applied during non-invasive support may be inaccurate and overestimated due to leakage and gas mixture in the upper airways, bearing risks for bias when compared to intubated patients.

## 4. Overview of ARDS definitions used in clinical trials involving non-intubated patients

Various authors and study groups have employed different methods to address the issue of measuring the PaO_2_/FiO_2_ ratio to define ARDS in non-intubated patients. In several publications, the term ARDS has been omitted in favor of the more general description “acute (hypoxemic) respiratory failure” (30, 59). Data from such studies have been implemented in guidelines for the management of ARDS (55). One guideline on NIV resolved to cluster ARDS, pneumonia, and hypoxemic respiratory failure in one collective category (11).

A significant body of evidence that – partially – still informs guidelines, originates from the era of the AECC definition (8, 15). Other studies applied the definition of ALI or the AECC criteria in their patient selection after the publication of the Berlin criteria, thus circumventing the issue of a PEEP requirement (60).

Formally, the Berlin criteria only explicitly acknowledge a diagnosis of ARDS in patients receiving non-invasive respiratory support in the context of mild disease (4). A methodologic approach to fully comply with the Berlin criteria in all aspects except for this limitation was employed in a trial assessing early prone positioning in patients with moderate to severe ARDS on HFNO and NIV. To determine disease severity, patients received an initial treatment with PEEP of 5 cmH_2_O *via* NIV facemask for 30 min (F_i_O_2_ 0.5) (12). A similar strategy for diagnosis and stratification was used in the LUNG-SAFE study (13, 61), a trial focused on a predefined sequence of conventional oxygen therapy, HFNO and NIV in patients with AHRF and ARDS (62), and a clinical study evaluating predictors of NIV failure (16).

Conversely, in a recent study assessing the effect of NIV on intubation rate, as compared to conventional oxygen therapy *via* Venturi mask in early mild pneumonia-associated ARDS, the PEEP criterion of the (otherwise employed) Berlin definition was consciously omitted (63). This strategy was chosen to avoid the dilemma of switching the control group back from a system delivering PEEP to a Venturi mask. The same method was employed in two recent studies evaluating intubation rates in patients with HFNO in acute respiratory failure and, specifically, the subsets of ARDS and COVID-19 ARDS (58).

Of note, Hultström and colleagues reported a poor predictive value of the P_a_O_2_/F_i_O_2_ ratio on HFNO for ARDS severity assessed on MV in a cohort of COVID-19 patients (64). While the ratios were similar when NIV and MV were compared, most patients showed improvements of the P_a_O_2_/F_i_O_2_ ratio when switched to MV. This may also explain the remarkably low mortality of seemingly moderate to severe ARDS found in a trial on HFNO in the early months of the pandemic (58).

## 5. Current research gaps and perspectives

**Figure 1:**
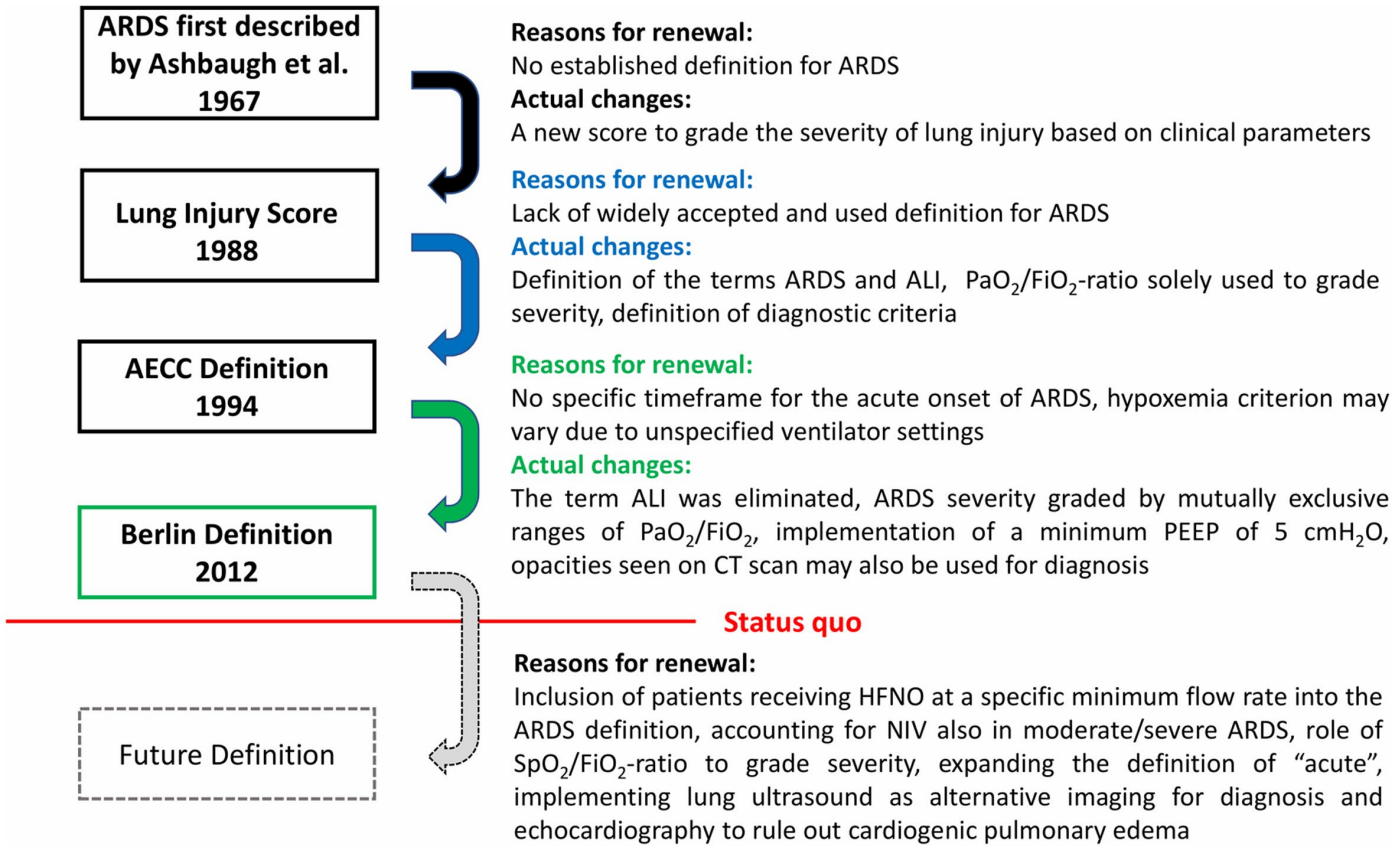
Questions arising in the development of ARDS definitions and future challenges. Multiple adaptations of the definition of acute respiratory distress syndrome (ARDS) have been implemented since its first description in 1967. Further changes leading to a new ARDS definition may arise from a growing body of evidence and constant evolution of clinical practice in acute respiratory failure. ALI, acute lung injury; PEEP, positive end-expiratory pressure; HFNO, high flow nasal oxygen therapy; NIV, non-invasive ventilation.

The history of the ARDS definition is one of repeated reappraisal of evidence and reevaluation of contemporary methods. This section addresses current knowledge gaps that may be factored into a future revised definition of ARDS (Figure 1).

The dilemma of valid ARDS diagnosis in patients receiving HFNO has been addressed before, and has become more evident during the COVID-19 pandemic and the associated scarcity of resources (65). The latter has led to the widespread use of non-invasive ventilatory support in patients who would normally have been intubated and mechanically ventilated (14, 17, 18, 28, 58). Some authors have therefore suggested an expansion of the Berlin Definition to include patients receiving HFNO at a flow rate of at least 30 l/min (66). Considering experimental data showing that a median PEEP of ≥5 cmH_2_O was not achieved at a flow rate of 40 l/min, but could be achieved at a flow rate of 60 l/min in most patients, any such expansion would warrant discussion of this cutoff (24).

Evidence on the rationale for assessing ARDS severity on HFNO is equivocal. A switch from HFNO to MV may lead to significant improvements of the P_a_O_2_/F_i_O_2_ ratio in the range of 50–150 mmHg and thus, to a different classification of ARDS severity (64). Whether or not this observation is entirely linked to constant and direct application of PEEP with MV or lack thereof as well as inaccurate FiO_2_ under HFNO is uncertain. An additional finding of a trial assessing the pharyngeal end expiratory pressure under HFNO and CPAP was that a CPAP set to 4 cmH_2_O achieved a median end-expiratory pressure of 3.1 cmH_2_O (24). This observation may raise doubt as to whether a tight-fitting CPAP mask set to 5 cmH_2_O as described in the current ARDS definition could indeed achieve that pressure.

Other novel therapeutic approaches may also warrant reconsideration of our current definition of ARDS. While prone positioning of intubated patients with severe ARDS is now considered standard of care due to reduced mortality (67), awake prone positioning in non-intubated patients has more recently emerged as a promising therapeutic option during the COVID-19 pandemic (12, 68). Awake prone positioning has recently been shown to reduce mortality and improve oxygenation, although effects on other clinically relevant outcomes are less clear (69). In the light of these findings and the increasing use of this practice, future definitions of ARDS may be required to take such interventions into account in interpreting a P_a_O_2_/F_i_O_2_ ratio. For example, future research may address the question of whether severity is best defined according to baseline oxygenation indices or “best oxygenation achieved,” using a bundle of such maneuvers and non-invasive respiratory support methods.

The definition of ARDS severity based on the P_a_O_2_/F_i_O_2_ ratio alone provides a good predictive value for mortality, ranging from 27 to 32 and 45% in mild, moderate, and severe ARDS, respectively (4). This singular index may, however, only reflect one layer of a multi-facetted pathophysiology. For example, right ventricular (RV) dysfunction, a feature that frequently accompanies ARDS, is not reflected in the current classification, and has been linked to worse outcomes (70–72). Several mechanisms have been proposed how the RV is stressed in ARDS, even in patients receiving lung protective ventilation. Limiting the driving pressure appears to protect the RV (73), while permissive hypercapnia and conservative oxygenation targets may promote pulmonary vasoconstriction. High transpulmonary pressures caused by MV may lead to compression of the pulmonary vasculature, and vascular obstruction due to coagulopathy might further increase RV afterload (74). Consecutively, reduced preload and cardiac output in the systemic circulation as well as venous congestion, leading to edema and a reduced arterio-venous pressure gradient, may worsen systemic perfusion and contribute to multiorgan dysfunction in ARDS (75). Therefore, accounting for RV failure according to clearly validated criteria in future ARDS definitions with regards to disease severity may be prudent. Further clarification of the clinical relevance of the heart-lung-interplay in ARDS may broaden our understanding of potential benefits of early initiation of interventions, such as prone positioning, inhaled pulmonary vasodilators and extracorporeal life support and in this subgroup of patients (74, 76).

## 6. Conclusion

Further research in the respiratory care of ARDS outside invasive MV is required. The rapid developments in this field over the last years paired with, and accelerated by, the immense burden of disease of COVID-19-associated ARDS have exposed potential gaps in the applicability of our current ARDS definition. Addressing this issue may lead to faster identification and guideline-directed treatment of ARDS, ultimately offering the potential to improve outcomes. Thus, dedicated research informing a possible evidence-based expansion or update of the Berlin Definition is needed.

## Author contributions

MZ and MW drafted the manuscript. FK and MW prepared the figure. MW prepared the table. SK, FK, RU, and KK assisted with literature search and reviewed and edited the manuscript. KK supervised the writing process. All authors contributed to the article and approved the submitted version.

## Funding

KK reports a grant from Apeptico GmbH that was used in part for covering open access expenses. The funder was not involved in the design of the article, the collection of literature sources, the interpretation of data, the writing of this article, or the decision to submit it for publication.

## Conflict of interest

KK reports travel expenses from Biotest GmbH and grants from Apeptico GmbH, Biotest GmbH, Alterras Therapeutics GmbH, and Bayer AG. RU reports travel expenses and honoraria from Biotest GmbH, grants from CCORE, a patent (EP 151897774, minimally invasive liquid lung), and holds equity in CCORE Technology, a medical device company that develops minimally invasive blood purification devices.

The remaining authors declare that the research was conducted in the absence of any commercial or financial relationships that could be construed as a potential conflict of interest.

## Publisher’s note

All claims expressed in this article are solely those of the authors and do not necessarily represent those of their affiliated organizations, or those of the publisher, the editors and the reviewers. Any product that may be evaluated in this article, or claim that may be made by its manufacturer, is not guaranteed or endorsed by the publisher.
